# The Role of Environmental Factors on Sleep Patterns and School Performance in Adolescents

**DOI:** 10.3389/fpsyg.2015.01717

**Published:** 2015-12-01

**Authors:** Dagmara Dimitriou, Frances Le Cornu Knight, Patrick Milton

**Affiliations:** Lifespan Learning and Sleep Laboratory, Department of Psychology and Human Development, UCL Institute of EducationLondon, UK

**Keywords:** sleep, adolescent, learning, stimulants, sleep deprivation

## Abstract

**Background:** Modern life, with its many distractions, is seeing sleep quantity and quality decline during adolescence. This is a concern as research persuasively demonstrates the negative impact of reduced sleep on academic achievement, both in terms of learning and behavior.

**Aims:** This study examined the relationship between sleep and school functioning in adolescence, with a focus on environmental factors that might mediate this relationship.

**Sample and Method:** Forty-seven adolescents took part. Sleep was measured using the School Sleep Habits Survey (SSHS) and a sleep diary. School records of year grade point averages provided a measure of academic achievement. Raven's Standard Progressive Matrices gave a measure of general cognitive processing. Environmental sleep factors falling into three groups, namely, stimulant consumption, media use and exercise, were measured using a self-report questionnaire.

**Results:** An average of 7.08 h of sleep was reported. Correlations revealed that Total sleep time (TST) and bedtimes on weekdays were strongly associated with academic achievement. Morning/eveningness and sleep/wake behavior problems had a strong relationship with performance on the Ravens. Stimulant consumption and media use before bed revealed strong relationships with TST and bedtimes on weekdays. Crucially, mediation analyses confirmed that both caffeine consumption and electronic media use before bedtime were negatively associated with academic performance, via the mediating pathway by affecting sleep. Exercise was not associated with any of the sleep variables, but was associated with better academic performance.

**Conclusion:** The current findings highlight that, now more than ever, parents, schools and policy makers must be aware of the negative effects of caffeinated substances marketed to students, and electronic media use on their sleep habits. Our findings suggest that targeting caffeine consumption and electronic media use before bed may represent effective routes in alleviating modern teenage sleep debt, and in turn enhancing academic performance.

## Introduction

Sleep plays a vital role in healthy development through childhood and adolescence. It supports physical and neurobiological development (Picchioni et al., 2014), as well as facilitating academic learning (Taras and Potts-Datema, 2005), and cognitive functioning. Optimal sleep quality and duration has been associated with better academic performance and behavioral regulation throughout lifespan development (Fredriksen et al., 2004). However, the modern world is increasingly associated with attractive social and media distractors, which are accessible 24 h. Emerging research highlights the negative effect of computer games and electronic media exposure, on sleep/wake regulation, sleep quality, and duration (e.g., Dworak et al., 2007; Garmy et al., 2012; Peiro-Velert et al., 2014). This paper explores how environmental factors commonly associated with modern adolescent lifestyles (such as electronic media use, stimulant consumption) and exercise relate to the established relationship between sleep and academic performance.

Sleep is essential for processes of memory consolidation, learning capacity, and academic performance (for review see Curcio et al., 2006). For example, Gruber et al. (2014) have shown that sleep efficiency is highly related to academic performance on the core subjects English and Maths (as well as French as a second language) 7–11 year olds. Likewise, Gillen-O'Neel et al. (2013) found that 13–16 year-olds who sacrifice habitual sleep in favor of additional study, hinder their capacity to understand class material, and struggle more on assignments or tests the subsequent day. Gomes et al. (2011), suggest that this continues on into early adulthood. The authors find that self-reported sleep quality and quantity are the main predictors of academic performance in full-time undergraduate students aged between 17 and 25 years. Studies experimentally restricting sleep, thus allowing insight into the direction of this relationship, confirm that sleep deprivation leads to a decrease in teacher-reported attention and academic achievement (Fallone et al., 2000), neurobehavioral functioning (Sadeh et al., 2003), and educational task performance.

Importantly, the negative impact of poor sleep on cognitive and academic functioning, is not always matched by an overt realization of this fact by the students themselves. For instance, Pilcher and Walters (1997) assessed the impact of sleep deprivation on cognitive performance. Forty-four college students were asked to complete the Watson-Glaser Critical Thinking Appraisal, after either 8 h of sleep or 24 h of sleep deprivation. The sleep deprived group performed significantly worse than those who slept, and yet rated their concentration, attention and estimated performance more favorably. In other words, the objective deterioration in cognitive function is not partnered with a subjective awareness of the decline. Therefore, adolescents may not have insight into the negative impact of sleep loss and as such will have little motivation to alter bad habits.

Despite the substantiated link between insufficient sleep and poor academic performance, the modern-day teenager is getting less and less sleep (Wolfson and Carskadon, 2003; Curcio et al., 2006). Matricciani et al. (2011) performed a systematic review of sleep data from 690,747 children and adolescents, and found that sleep duration has consistently decreased over the past century. Eaton et al. (2010) found that 69% of American teenagers sleep less than 7 h per night; an hour and a half less than recommended (Carskadon et al., 2001). Recent research suggests that the majority of adolescents are at risk of delayed circadian patterns owing to habitually later bedtimes (Wolfson and Carskadon, 1998; Crabtree and Witcher, 2008). Indeed, Carskadon et al. (2006) suggest that habitual weeknight sleep loss gets worse as individuals advance through their teenage years. In a survey of over 1500 11–18 year-olds, the authors report that total sleep time (TST) reduces dramatically from 8.4 to 6.9 h per night. And yet, when left to sleep uninterrupted in a darkened room mature adolescents consistently require more sleep than their younger counterparts (Carskadon et al., 1980). A number of environmental factors that have been considered in explaining the increasing sleep debt in adolescents.

One potential explanation for teenage sleep debt is the introduction of television sets, games consoles, mobile phones, laptops, and tablets into the bedroom (Johnson et al., 2004; Eggermont and Van den Bulck, 2006; Shochat et al., 2010). In the US in 2013, 82% of young people aged 12–17 years owned either a mobile phone or a tablet, and 74% of those had access to the internet (Madden et al., 2013). As adolescents have greater access to technology use in their bedrooms, several studies have noted the link between this modern day phenomenon and the reducing hours of sleep (for review see Cain and Gradisar, 2010). For instance, Adam et al. (2007) report that excessive media consumption before bedtime leads to delayed sleep onset and shorter TST. Likewise, Dworak et al. (2007) report that television and video use before bed negatively impacts on sleep quality and cognitive functioning. Even those adolescents who report using music and television for the purpose of aiding sleep, sleep fewer hours and are significantly more tired (Eggermont and Van den Bulck, 2006).

The consumption of stimulants, such as caffeine, nicotine, and alcohol has also been linked to reduced sleep quantity and quality in adolescents (Johnson et al., 2006; Lohsoonthorn et al., 2013). Adolescents who consume higher quantities of caffeinated drinks are twice as likely to experience sleep problems, both in terms of quality and quantity (Wright et al., 1997), as well as being more likely to report daytime sleepiness (Calamaro et al., 2009). Likewise, nicotine and alcohol use during adolescence has been associated with sleep problems (Johnson et al., 2006; Cox et al., 2007). Cigarette smokers are significantly more likely than non-smokers to report difficulty in maintaining sleep, leading to increased daytime sleepiness (Phillips and Danner, 1995) and reduced daytime functioning (Lohsoonthorn et al., 2013). Given that sleep is related to academic performance, and that stimulant use disrupts sleep, it is reasonable to assert that stimulant use may indirectly affect academic performance by negatively impacting on sleep quality and/or quantity (Johnson et al., 2006; Singleton and Wolfson, 2009).

The final environmental factor considered here is the beneficial effect of exercise on sleep and academic performance. Whilst many professionals and non-professionals believe in the beneficial effects of exercise on sleep, far fewer studies have been performed. Athletes report better sleep quality, fewer night-time awakenings, and shorter sleep onset latency, as well as less daytime sleepiness and increased concentration during the day compared to controls (Brand et al., 2010). In the non-elite athlete too, evidence suggests that aerobic exercise has the effect of deepening sleep. In typical adolescents, those who exercise more frequently (averaging 8.5 h a week) spend more of their night in slow wave sleep, and less time in light or REM sleep, than low exercisers (2 h a week; Brand et al., 2010). Therefore, exercise may play a key role in ensuring healthy sleep patterns, by increasing the sleep stage that optimizes academic performance.

The current study examines if stimulant consumption, electronic media use and exercise are related to sleep and academic performance. Whilst the influence of such environmental factors on sleep has been studied in teens, the specific knock-on effect that this has on academic performance is not well determined. We assessed academic performance (using yearly grade point averages; GPA), and fluid intelligence score (using Raven's Progressive Matrices). Sleep was assessed using the School Sleep Habits Survey (SSHS). Finally, the environmental factors discussed above were assessed using a self-report questionnaire. The use of standard GPA and self-report questionnaires, allows a snapshot of adolescent behaviors without disrupting daily living. We believe this to be an important avenue of research, as it represents a viable route to alleviating sleep debt in teens by educating people about the damaging effect casual daytime behaviors can have on sleep and on academic performance more globally.

## Methods

### Participants

Forty-eight 16–19-year-old (19 male/28 female; mean age 17 years) students were recruited through an independent sixth form college in central London. All participants were white, spoke English as their first language and were recruited from a region of middle-to-high socioeconomic status. On the basis of a medical questionnaire, one participant was excluded due to moderate asthma (which may disturb sleep). No further medical conditions were reported. Ethical approval was granted by the UCL-IoE Ethics Committee and the Principal of the college. All participants gave written informed consent and parental consent prior to taking part.

### Measures

#### The school sleep habits survey

The School Sleep Habits Survey (SSHS; Wolfson and Carskadon, 1998; Wolfson et al., 2003) The SSHS is a 63 items questionnaire designed to measure sleep related behavior and daytime functioning in high school students. Items on the questionnaire combine to give measures of habitual weekday and weekend sleep profiles, sleepiness, sleep/wake problem behaviors, circadian preference (morningness/eveningness; M/E) as well as a depressive mood scale.

#### Sleep diary

All participants completed a simple sleep diary during mock week. Participants recorded bed- and rise-times, and the frequency and duration of nighttime awakenings and daytime naps. Only 40 sleep diaries were returned at the end of mock week.

#### Year grade point average (year GPA)

Percentage scores were taken from the college's database of monthly assessments which covered recent course content. The mean GPA was calculated for the autumn and spring terms.

#### Raven's standard progressive matrices plus (SPM)

The Raven's SPM is a cognitive assessment of non-verbal reasoning standardized for pupils aged 7–18 years. The test was administered during students' free periods in the 3 weeks prior to mock week.

#### Background lifestyle and medical questionnaire (BLMQ)

This assessed lifestyle habits including: smoking, and energy drink, caffeine, and alcohol consumption; television viewing, video game and social media use; and exercise. Medical questions were aimed at elucidating health problems that are known to affect sleep such as, behavioral disorders, problems with adenoids and tonsils, and poorly controlled asthma.

### Procedure

Participants were given the BLMQ followed by the SSHS to complete under the teacher's supervision. Students were then required to complete the Raven's SPM under the supervision of the researcher within 3 weeks after this date. Sleep diaries were distributed for completion at the beginning of the third week; an email reminder was sent out 1 week before the required start date. Monthly assessment records were made available to the researcher by the school, from which year GPA scores were calculated.

### Data analysis

Data analysis took two phases. Firstly, in order to confirm the relationships between the SSHS sleep variables, environmental factors, and academic functioning (year GPA), a series of partial Pearson's correlations were performed, controlling for age. From these analyses, three composite environmental factors [Electronic Media Before Bed (EMBB), Caffeine Consumption and Exercise)] and one sleep variable [Total Sleep Time; (TST)] were identified for further analysis owing to their consistently strong relationships with year GPA and sleep variables.

In the second phase of analysis three separate mediation analyses were performed, in order to elucidate whether the key environmental factors influenced academic performance via the route of affecting sleep. All mediation regression used TST on weekdays as the mediating variable and, owing to the results of the correlations, non-verbal IQ was controlled for using the Ravens SPM. First, we explored the relationship between exercise and academic performance, using TST weekdays as a mediator. Second, we explored the relationship between caffeine consumption and academic performance, again using sleep as the mediator. The third analysis explored the relationship between technology use 30 min before bed and academic performance, again using sleep as the mediator.

## Results

SSHS and background questionnaires were returned from all students. Sleep diaries were returned from 40 students. For descriptive statistics of sleep and school functioning variables, see Tables 1–3. Sleep variables (SSHS), environmental factors and Year GPA were first analyzed using partial Pearson's Correlations, controlling for non-verbal IQ. The results of these correlations are reported in Table 4. For all results, correlations greater than ±0.50 were taken as representing strong associations, and those between ±0.30 and ±0.50 were taken as moderate. Correlations below ±0.30 were considered weak and therefore are not discussed in the body of the text.

**Table 1:** **Mean and standard deviations of sleep measures from SSHS data**.

	***N***	**Minimum**	**Maximum**	**Mean**	**Std. deviation**
Bedtime weekdays	47	22:00	4:00	23:37	1:13
Wake up weekdays	47	5:00	09:30	7:05	0:49
TST weekdays	47	2 h	9 h 40	7 h 08	0 h 09
Bedtime weekends	47	22:30	07:00	01:01	1:37
Wake up weekends	47	7:00	16:00	10:12	1:38
TST weekends	47	5 h	11 h 30	9 h 04	1 h 39
Weekend sleep lag	47	−1 h 30	5 h 30	1 h 55	1 h 33
Sleepiness	47	10.00	40.00	18.72	7.20
Sleep/wake behavior problems	47	12.00	40.00	23.98	7.80
M/E scale	47	13.00	39.00	24.43	7.38

**Table 2:** **Mean and standard deviations of mock week sleep measures from sleep diary**.

	***N***	**Minimum**	**Maximum**	**Mean**	**Std. deviation**
Mock Bedtime	40	22:39	01:01	23:48	0:35
Mock wake up	40	06:45	09:42	07:45	0:39
Mock TST	40	6 h 14	9 h 45	7 h 57	0 h 54

**Table 3:** **Mean and standard deviations of school functioning variables**.

	***N***	**Minimum**	**Maximum**	**Mean**	**Std. deviation**
Year GPA (%)	47	46.80	94.34	72.38	12.52
Raven's SPM score	47	70.00	120.00	97.87	11.50
Mock GPA (%)	47	48.85	92.87	74.36	11.08
Depressive mood score (SSHS)	47	6.00	41.00	11.43	5.40

**Table 4:** **Correlation coefficients for SSHS sleep variables (columns), environmental factors (rows), and year GPA**.

	**Pearson correlation coefficients**											
	**Year GPA**	**Bed time weekdays**	**Wake up weekdays**	**TST weekdays**	**Bed time weekends**	**Wake up weekends**	**TST weekends**	**Weekend sleep lag**	**Sleepiness**	**Sleep/wake behavior problems**	**Depression scale**	**M/E scale**
Year GPA	1.00	−0.39^*^	0.10	0.43^**^	−0.51^**^	−0.11	0.22	−0.16	0.22	−0.10	0.08	0.23
Exercise hours per week	0.41^**^	−0.22	0.23	0.22	−0.32^*^	−0.01	0.18	−0.01	−0.11	−0.17	0.01	−0.06
Smoke yes/no	0.14	0.19	0.21	−0.20	0.03	0.08	0.03	0.23	−0.13	−0.16	0.02	−0.26
How many cigarettes smoked per day	−0.15	0.02	−0.26	−0.05	−0.02	−0.10	−0.17	−0.15	0.11	0.13	0.04	0.14
How often alcohol is consumed	−0.08	0.34^*^	0.00	−0.27	0.10	−0.04	−0.23	−0.02	0.13	0.31	0.04	−0.07
How many units during a drinking session?	0.01	0.11	−0.11	−0.08	−0.12	−0.07	0.08	0.17	0.17	−0.15	−0.08	−0.13
How often energy drinks are consumed	−0.42^**^	0.56^**^	−0.04	−0.53^**^	0.20	0.05	−0.26	0.21	−0.16	0.28	0.04	−0.30
How often coffee is consumed	−0.34^*^	0.61^**^	0.12	−0.33^*^	0.15	−0.07	−0.07	0.23	−0.01	0.01	−0.04	−0.25
Combined caffeinated consumed	−0.45^**^	0.70^**^	0.06	−0.48^**^	0.17	−0.03	−0.21	0.21	−0.09	0.16	−0.01	−0.30
TV weekdays	−0.21	0.34^*^	−0.10	−0.35^*^	0.07	−0.13	−0.19	0.11	−0.35^*^	0.15	0.13	0.01
TV weekends	−0.25	0.26	−0.08	−0.29	0.12	−0.21	−0.21	0.03	−0.25	0.09	0.09	0.13
TV 30 min before bed	−0.44^**^	0.73^**^	0.04	−0.54^**^	0.21	−0.07	−0.16	0.33^*^	−0.25	−0.07	0.02	−0.28
Social media weekdays	−0.22	0.03	−0.26	−0.19	0.35^*^	−0.16	−0.23	−0.09	0.06	0.02	−0.07	0.35^*^
Social media weekends	0.20	−0.16	−0.22	0.04	−0.11	−0.21	0.05	0.02	−0.06	−0.22	−0.21	0.21
Social media 30 min before bed	−0.44^**^	0.71^**^	−0.06	−0.52^**^	0.19	−0.07	−0.19	0.28	−0.17	0.07	−0.09	−0.35^*^
TV in bedroom	0.02	−0.21	−0.07	0.14	0.13	0.11	−0.04	−0.18	0.04	−0.06	−0.40	0.00

Significant correlations are highlighted in bold with asterisks representing significance levels; ^*^p < 0.05, ^**^p < 0.01 (1-tailed).

### Exploratory correlations

Exploratory Pearson's correlations were performed between GPA, Raven's SPM, SHSS and BLMQ responses, controlling for age (see Table 4). Associations between SSHS responses and GPA revealed moderate and strong correlations between *GPA* scores and *Weekday Bedtimes, Weekend Bedtimes* and *TST on weekdays*, such that better school results were associated with earlier bedtimes and more hours of sleep a night. GPA was also moderately negatively correlated with *Sleep/Wake Problem Behaviors*, and positively with *Morningness/Eveningness Scale*. *GPA* correlated with a number of items on the BLMQ. Crucially, *GPA* revealed a moderate positive relationship with *Exercise*, such that more exercise was associated with better academic performance. *Caffeine consumption* (across three variables) showed a moderate to strong negative relationship with *GPA*; the more caffeine consumed, the worse the year GPA. Likewise, the use of both *TV* and *social media 30 min before bed* revealed moderate negative relationships with *GPA*. *Raven's SPM* correlated positively with *GPA* (strong effect), *TST weekdays* (moderate), and *Morningness/Eveningness Scale*, and negatively with *Sleep/Wake Behavior Problems*, and smoking habits and alcohol, such that those who smoked more and drank more alcohol had lower Raven's scores.

The most consistent relationships between the BLMQ and SSHS variables were of *Weekday Bedtimes* and *TST on weekdays*, each revealing moderate to strong relationships with six of the 15 identified environmental sleep factors respectively. These fell into two main clusters: caffeine consumption (across three variables; energy drink, coffee, and combined caffeine consumption) and technology use 30 min before bed (across two variables; *TV*, and *social media use 30 min before bed*). Both caffeine consumption and technology use before bed were associated with later bedtimes and reduced TST. As a result of these correlations, a new variable, *EMBB*, was computed which combined TV and social media use 30 min before bed. *EMBB, Combined Caffeine Consumption* and *Exercise* were analyzed further.

### Mediated regressions

Exercise, caffeine consumption and use of technology before bed all correlated with year GPA. In order to elucidate whether these environmental factors predicted year GPA directly, or indirectly via the route of sleep, three separate mediation analyses were conducted. In order to reduce the influence of baseline IQ (which correlates both with TST weekdays and GPA), Ravens SPM was used as a control variable in each mediated regression.

**Figure 1:**
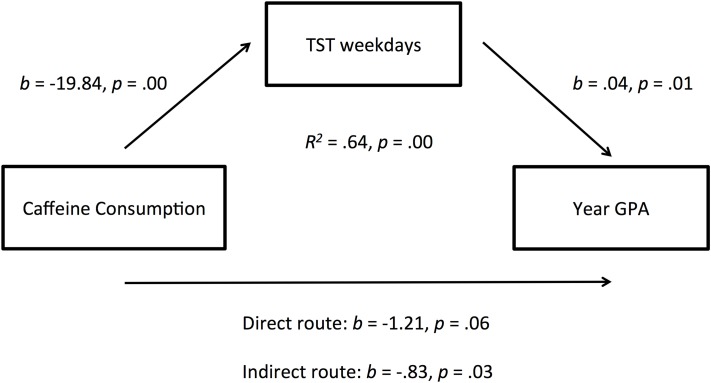
**Regression coefficients, and significance levels, for the relationship between caffeine consumption and year GPA as mediated by total sleep time on weekdays**.

**Figure 2:**
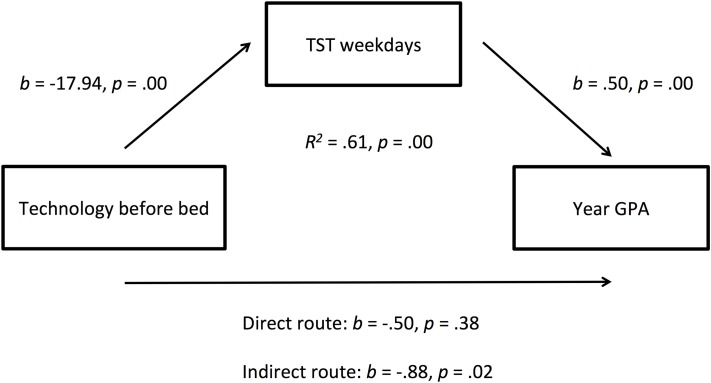
**Regression coefficients, and significance levels, for the relationship between technology use before bed and year GPA as mediated by total sleep time on weekdays**.

The first examined the relationship between *Exercise* and *Year GPA*, with *TST on Weekdays* as a mediator, and controlling for non-verbal IQ using the *Ravens SPM*. An *R*^2^-value showed that the overall model explained 61.71% of the variation in Year GPA (*R*^2^ = 0.62, *p* = 0.001), however scrutinizing the regression model further this was the result of a strong relationship between TST weekdays and non-verbal IQ on academic performance. Exercise did not return a significant direct effect on year GPA in this mediated regression model. The second mediated regression examined the relationship between *Combined Caffeine Consumption* and *Year GPA* via the mediated pathway of *TST on Weekdays*, again controlling for non-verbal IQ (See Figure 1). There was a significant indirect effect of caffeine consumption on year GPA via TST on weekdays, *b* = −0.83, BCa CI [−1.86, −0.27], *p* = 0.02. Using a *completely standardized effect size* (Preacher and Kelley, 2011) this represents a large effect size, *ab*_*cs*_ = −0.19, 95% BCa CI [−0.40, −0.07]. Finally, the third mediated regression was conducted, this time examining the use of *EMBB* and *Year GPA*, with *TST on weekdays* as the mediator, and controlling for non-verbal IQ. There was a significant indirect effect of *EMBB* on *GPA* via sleep, *b* = −0.88, BCa CI [−1.80, −.24], *p* = 0.01, suggesting that electronic media use before bed reduces academic performance by decreasing sleep on weeknights (Figure 2). Again, this represented a large effect size *ab*_*cs*_ = −0.23, 95% BCa CI [−0.47, −0.07]. Although our sample size is rather small the narrow age range and magnitude of the effect sizes of α and β pathways are relatively good.

## Discussion

In the current study, we examined how modern-day lifestyle factors affect the relationship between sleep and academic performance in adolescents. We report three key findings. Firstly, in line with previous research, adolescents in our study report achieving less than the recommended 9–10 h sleep per night (Wolfson and Carskadon, 1998; Crabtree and Witcher, 2008). Secondly, weekday bedtimes, and total sleep times are strongly related to academic achievement, namely, later bedtimes and fewer hours sleep are associated with reduced academic performance (Wolfson and Carskadon, 1998; Curcio et al., 2006; Lowry et al., 2010). Finally, stimulant consumption and electronic media use 30 min before bed are negatively associated with academic performance via the mediated pathway, by negatively affecting adolescent sleep. No association between sleep and exercise was found, however exercise was positively associated with academic performance.

The adolescents in our study achieved on average 7 h 08 min of sleep on a weeknight and their average bedtime was at 23:37. These findings confirm previous reports of an increasing sleep debt problem in adolescents (Wolfson and Carskadon, 1998; Crabtree and Witcher, 2008; Moore and Meltzer, 2008; Noland et al., 2009; Taylor and Bramoweth, 2010). Furthermore, weekday bedtimes and TST were most strongly associated with academic performance; those who achieved less sleep (and had later bedtimes) produced worse academic results. This corroborates reports from countries around the world (e.g., Canada, Galambos et al., 2009; China, Liu et al., 2008; Israel, Shochat et al., 2010; Italy, Curcio et al., 2006; Thailand, Lohsoonthorn et al., 2013; US, Wolfson and Carskadon, 1998; Lowry et al., 2010). It highlights the necessity for ongoing research into adolescent sleep debt in order to establish the exact causal factors, and to increase awareness about the benefits of optimal number of sleep on our cognitive and behavioral functioning.

How each sleep characteristic relates to our performance measures suggests potentially different roles for sleep quantity and sleep quality on daily functioning. Greater TST and earlier bedtimes (measures of sleep quantity) were most strongly correlated with better academic results. Conversely, morning/eveningness and sleep/wake behavior problems (more representative of sleep quality) were more strongly associated with performance on Ravens matrices, followed by year GPA. This may indicate that sleep quantity is more closely related to academic performance, whilst sleep quality being more closely related to global cognitive processing (Meijer et al., 2000). Indeed, prior research suggests that sleep quality, rather than quantity, impacts higher order cognitive processing (for review see, Killgore, 2010), working memory and memory consolidation (for review see Kopasz et al., 2010). The fact that sleep quality is associated Ravens Matrices scores and with year GPAs follows normal trajectory that more effective cognitive processing overall would engender better academic performance.

In terms of the impact of reduced sleep quantity, previous research is mixed. Some authors report that sleep restriction negatively impacts on adolescent academic performance (Fallone et al., 2000, 2005) and cognitive processing (Sadeh et al., 2003; Ashworth et al., 2014). Others suggest adolescents are more resilient to sleep restriction (Carskadon et al., 1981; Kopasz et al., 2010). Voderholzer et al. (2011) showed that later in adolescence, individuals have the capacity to up-regulate slow-wave sleep (which is particularly important for memory consolidation) following sleep restriction, making the sleep that they did achieve more efficient. Like others, we found that habitually lower amounts of sleep were associated with lower GPA (Wolfson and Carskadon, 1998; Eliasson et al., 2002, 2010). More specifically, we also found that later bedtimes and lower TST during a week of examinations were strongly associated with poorer exam scores. Whilst the nature of correlational research means we cannot infer a causal direction, the work of Fallone and colleagues (Fallone et al., 2000, 2001, 2005; see also Sadeh et al., 2003) indicates that the direction of this relationship is such that reduced sleep negatively impacts on academic performance.

Finally, we examined several lifestyle factors that may be influencing the modern-day adolescents to achieve fewer than the recommended hours of sleep. We found that caffeine consumption, and media use 30 min before bed were strongly associated with weekday bedtimes and TST. More specifically, both energy drink and coffee consumption were strongly associated with later bedtimes. These findings are in line with early research (Phillips and Danner, 1995; Hofman and Steenhof, 1997; Wright et al., 1997) demonstrating the negative impact of such substances on sleep quantity. More recent studies report, a negative association between stimulant consumption and sleep quality (alcohol, Galambos et al., 2009; Kenney et al., 2012; energy and caffeinated drinks, Lohsoonthorn et al., 2013; nicotine, Zunhammer et al., 2014). Of great concern is the finding that there is a knock-on effect of on academic performance. Here we provide preliminary evidence that caffeine consumption in adolescence reduces sleep, which in turn negatively affects academic performance.

We also report that watching TV and/or using social media 30 min before bed was strongly associated with later bedtimes and reduced TST midweek. This finding corroborates others suggesting that the modern-day dependence on electronic media has a negative effect on adolescent sleep patterns (Shochat et al., 2010). There are a number of mechanisms that may be at play in the association between media exposure and sleep (Cain and Gradisar, 2010). It is possible that using media before bed, over time displaces the natural circadian rhythm as an extra half an hour watching a programme, changes circadian clock which becomes entrained by the new later sleep pattern. This has the potential to become gradually later and later. Exciting television shows and even social media may serve to increase physiological arousal making it more difficult to sleep. This has certainly been evidenced with playing computer games before bedtime (Hébert et al., 2005; Wang and Perry, 2006). Lastly, the bright light that emanates from television and computer screens, and specifically the electromagnetic radiation from tablets and more advanced mobile telephones, may serve to suppress melatonin levels (Higuchi et al., 2005; Wood et al., 2006), the hormone responsible for readying the body for sleep. In a systematic review of such effects, Cain and Gradisar (2010) urge researchers to develop guidelines on the nature and duration of electronic media use before bedtime to be made known to the general public. It is however possible that individuals who suffer from sleep onset delay use social media before bedtime. It is important to note that only late night media exposure has negative effect on sleep (Dworak et al., 2007) not media use *per se*.

In sum, we suggest that increasing awareness about the negative effects of stimulants and late night media use on sleep and consequently academic performance is now crucial. Lastly, complexity of studying sleep requires bringing together researchers from different disciplines in order to use multi-dimensional methods. Use of objective sleep measures such as actigraphy or polysomonograhy along with biological, educational and social factors ought to take methodological priority.

## Funding

None of the authors received at any time payment or services from a third party (government, commercial, private foundation, etc.) for any aspect of the submitted work.

### Conflict of interest statement

The authors declare that the research was conducted in the absence of any commercial or financial relationships that could be construed as a potential conflict of interest.

## References

[B1] Adam E. K. Snell E. K. Pendry P. (2007). Sleep timing and quantity in ecological and family context: a nationally representative time-diary study. J. Family Psychol. 21, 4. 10.1037/0893-3200.21.1.4 17371105

[B2] Ashworth A. Hill C. M. Karmiloff-Smith A. Dimitriou D. (2014). Sleep enhances memory consolidation in children. J. Sleep Res. 23, 302–308. 10.1111/jsr.12119 24329882

[B3] Brand S. Gerber M. Beck J. Hatzinger M. Pühse U. Holsboer-Trachsler E. (2010). High exercise levels are related to favorable sleep patterns and psychological functioning in adolescents: a comparison of athletes and controls. J. Adolesc. Health 46, 133–141. 10.1016/j.jadohealth.2009.06.018 20113919

[B4] Cain N. Gradisar M. (2010). Electronic media use and sleep in school-aged children and adolescents: a review. Sleep Med. 11, 735–742. 10.1016/j.sleep.2010.02.006 20673649

[B5] Calamaro C. J. Mason T. B. Ratcliffe S. J. (2009). Adolescents living the 24/7 lifestyle: effects of caffeine and technology on sleep duration and daytime functioning. Pediatrics 123, 1005–1010. 10.1542/peds.2008-3641 19482732

[B6] Carskadon M. A. Acebo C. Seifer R. (2001). Extended nights, sleep loss, and recovery sleep in adolescents. Arch. Ital. Biol. 139, 301–312. 11330207

[B7] Carskadon M. A. Harvey K. Dement W. C. (1981). Acute restriction of nocturnal sleep in children. Percept. Mot. Skills 53, 103–112. 10.2466/pms.1981.53.1.103

[B8] Carskadon M. A. Harvey K. Duke P. Anders T. F. Litt I. F. Dement W. C. (1980). Pubertal changes in daytime sleepiness. Sleep 2, 453–460. 7403744 10.1093/sleep/2.4.453

[B9] Carskadon M. A. Mindell J. Drake C. (2006). Infant and adolescent sleep. J. Sleep Res. 15, 41–43. 10.1111/j.1365-2869.2006.00540_20.x16490001

[B10] Cox R. G. Zhang L. Johnson W. D. Bender D. R. (2007). Academic performance and substance use: findings from a state survey of public high school students. J. School Health 77, 109–115. 10.1111/j.1746-1561.2007.00179.x 17302852

[B11] Crabtree V. M. Witcher L. A. (2008). Impact of sleep loss on children and adolescents, in Sleep and Psychiatric Disorders in Children and Adolescents, ed Ivanenko A. (New York, NY: Informa Healthcare USA, Inc.), 139–148.

[B12] Curcio G. Ferrara M. De Gennaro L. (2006). Sleep loss, learning capacity and academic performance. Sleep Med. Rev. 10, 323–337. 10.1016/j.smrv.2005.11.001 16564189

[B13] Dworak M. Schierl T. Bruns T. Strüder H. K. (2007). Impact of singular excessive computer game and television exposure on sleep patterns and memory performance of school-aged children. Pediatrics 120, 978–985. 10.1542/peds.2007-0476 17974734

[B14] Eaton D. K. McKnight-Eily L. R. Lowry R. Perry G. S. Presley-Cantrell L. Croft J. B. (2010). Prevalence of insufficient, borderline, and optimal hours of sleep among high school students-United States. J. Adolesc. Health 46, 399–401. 10.1016/j.jadohealth.2009.10.011 20307832

[B15] Eggermont S. Van den Bulck J. (2006). Nodding off or switching off? The use of popular media as a sleep aid in secondary-school children. J. Paediatr. Child Health 42, 428–433. 10.1111/j.1440-1754.2006.00892.x 16898880

[B16] Eliasson A. Eliasson A. King J. Gould B. Eliasson A. (2002). Association of sleep and academic performance. Sleep Breath. 6, 45–48. 10.1055/s-2002-23157 11917265

[B17] Eliasson A. H. Lettieri C. J. Eliasson A. H. (2010). Early to bed, early to rise! Sleep habits and academic performance in college students. Sleep Breath. 14, 71–75. 10.1007/s11325-009-0282-2 19603214

[B18] Fallone G. Acebo C. Arnedt J. T. Seifer R. Carskadon M. A. (2001). Effects of acute sleep restriction on behavior, sustained attention, and response inhibition in children. Percept. Mot. Skills 93, 213–229. 10.2466/pms.2001.93.1.213 11693688

[B19] Fallone G. Acebo C. Seifer R. Carskadon M. A. (2005). Experimental restriction of sleep opportunity in children: effects on teacher ratings. Sleep 28, 1561–1567. 16408416 10.1093/sleep/28.12.1561

[B20] Fallone G. Seifer R. Acebo C. Carskadon M. A. (2000). Prolonged sleep restriction in 11-and 12-year-old children: effects on behaviour, sleepiness, and mood. Sleep 23, 28.

[B21] Fredriksen K. Rhodes J. Reddy R. Way N. (2004). Sleepless in Chicago: tracking the effects of adolescent sleep loss during the middle school years. Child Dev. 75, 84–95. 10.1111/j.1467-8624.2004.00655.x 15015676

[B22] Galambos N. L. Dalton A. L. Maggs J. L. (2009). Losing sleep over it: daily variation in sleep quantity and quality in Canadian students' first semester of university. J. Res. Adolescence 19, 741–761. 10.1111/j.1532-7795.2009.00618.x

[B23] Garmy P. Nyberg P. Jakobsson U. (2012). Sleep and television and computer habits of Swedish school-age children. J. School Nurs. 28, 469–476. 10.1177/1059840512444133 22472633 PMC3698512

[B24] Gillen-O'Neel C. Huynh V. W. Fuligni A. J. (2013). To study or to sleep? The academic costs of extra studying at the expense of sleep. Child Dev. 84, 133–142. 10.1111/j.1467-8624.2012.01834.x 22906052

[B25] Gomes A. A. Tavares J. de Azevedo M. H. P. (2011). Sleep and academic performance in undergraduates: a multi-measure, multi-predictor approach. Chronobiol. Int. 28, 786–801. 10.3109/07420528.2011.606518 22080785

[B26] Gruber R. Somerville G. Enros P. Paquin S. Kestler M. Gillies-Poitras E. (2014). Sleep efficiency (but not sleep duration) of healthy school-age children is associated with grades in math and languages. Sleep Med. 15, 1517–1525. 10.1016/j.sleep.2014.08.009 25441747

[B27] Hébert S. Béland R. Dionne-Fournelle O. Crête M. Lupien S. J. (2005). Physiological stress response to video-game playing: the contribution of built-in music. Life Sci. 76, 2371–2380. 10.1016/j.lfs.2004.11.011 15748630

[B28] Higuchi S. Motohashi Y. Liu Y. Maeda A. (2005). Effects of playing a computer game using a bright display on pre-sleep physiological variables, sleep latency, slow wave sleep and REM sleep. J. Sleep Res. 14, 267–273. 10.1111/j.1365-2869.2005.00463.x 16120101

[B29] Hofman W. F. Steenhof L. (1997). Sleep characteristics of Dutch adolescents are related to school performance. Sleep Wake Res. 8, 51–55.

[B30] Johnson E. O. Roth T. Breslau N. (2006). The association of insomnia with anxiety disorders and depression: exploration of the direction of risk. J. Psychiatr. Res. 40, 700–708. 10.1016/j.jpsychires.2006.07.008 16978649

[B31] Johnson J. G. Cohen P. Kasen S. First M. B. Brook J. S. (2004). Association between television viewing and sleep problems during adolescence and early adulthood. Arch. Pediatr. Adolesc. Med. 158, 562–568. 10.1001/archpedi.158.6.562 15184220

[B32] Kenney S. R. LaBrie J. W. Hummer J. F. Pham A. T. (2012). Global sleep quality as a moderator of alcohol consumption and consequences in college students. Addict. Behav. 37, 507–512. 10.1016/j.addbeh.2012.01.006 22285119 PMC4329778

[B33] Killgore W. D. (2010). Effects of sleep deprivation on cognition. Prog. Brain Res. 185, 105–129. 10.1016/B978-0-444-53702-7.00007-5 21075236

[B34] Kopasz M. Loessl B. Hornyak M. Riemann D. Nissen C. Piosczyk H.. (2010). Sleep and memory in healthy children and adolescents–a critical review. Sleep Med. Rev. 14, 167–177. 10.1016/j.smrv.2009.10.006 20093053

[B35] Liu X. Zhao Z. Jia C. Buysse D. J. (2008). Sleep patterns and problems among Chinese adolescents. Pediatrics 121, 1165–1173. 10.1542/peds.2007-1464 18519486

[B36] Lohsoonthorn V. Khidir H. Casillas G. Lertmaharit S. Tadesse M. G. Pensuksan W.. (2013). Sleep quality and sleep patterns in relation to consumption of energy drinks, caffeinated beverages, and other stimulants among Thai college students. Sleep Breath. 17, 1017–1028. 10.1007/s11325-012-0792-1 23239460 PMC3621002

[B37] Lowry M. Dean K. Manders K. (2010). The link between sleep quantity and academic performance for the college student. Sentience 3, 16–19.

[B38] Madden M. Lenhart A. Cortesi S. Gasser U. (2013). Teens and Mobile Apps Privacy. Pew Internet and American Life Project. Available online at: http://pewinternet.org/Reports/2012/Teens-and-Privacy.aspx

[B39] Matricciani L. Olds T. Williams M. (2011). A review of evidence for the claim that children are sleeping less than in the past. Sleep 34, 651–659. 21532959 10.1093/sleep/34.5.651 PMC3079945

[B40] Meijer A. M. Habekothé H. T. Van Den Wittenboer G. L. (2000). Time in bed, quality of sleep and school functioning of children. J. Sleep Res. 9, 145–153. 10.1046/j.1365-2869.2000.00198.x 10849241

[B41] Moore M. Meltzer L. J. (2008). The sleepy adolescent: causes and consequences of sleepiness in teens. Paediatr. Respir. Rev. 9, 114–120. 10.1016/j.prrv.2008.01.001 18513671

[B42] Noland H. Price J. H. Dake J. Telljohann S. K. (2009). Adolescents' sleep behaviors and perceptions of sleep. J. School Health 79, 224–230. 10.1111/j.1746-1561.2009.00402.x 19341441

[B43] Peiró-Velert C. Valencia-Peris A. González L. M. García-Massó X. Serra-Añóv P. Devís-Devís J. (2014). Screen media usage, sleep time and academic performance in adolescents: clustering a self-organizing maps analysis. PLoS ONE 9:e99478. 10.1371/journal.pone.0099478 24941009 PMC4062405

[B44] Phillips B. A. Danner F. J. (1995). Cigarette smoking and sleep disturbance. Arch. Int. Med. 155, 734–737. 10.1001/archinte.1995.00430070088011 7695462

[B45] Picchioni D. Reith R. M. Nadel J. L. Smith C. B. (2014). Sleep, plasticity and the pathophysiology of neurodevelopmental disorders: the potential roles of protein synthesis and other cellular processes. Brain Sci. 4, 150–201. 10.3390/brainsci4010150 24839550 PMC4020186

[B46] Pilcher J. J. Walters A. S. (1997). How sleep deprivation affects psychological variables related to college students' cognitive performance. J. Am. Coll. Health 46, 121–126. 10.1080/07448489709595597 9394089

[B47] Preacher K. J. Kelley K. (2011). Effect size measures for mediation models: quantitative strategies for communicating indirect effects. Psychol. Methods 16, 93–115. 10.1037/a0022658 21500915

[B48] Sadeh A. Gruber R. Raviv A. (2003). The effects of sleep restriction and extension on school-age children: what a difference an hour makes. Child Dev. 74, 444–455. 10.1111/1467-8624.7402008 12705565

[B49] Shochat T. Flint-Bretler O. Tzischinsky O. (2010). Sleep patterns, electronic media exposure and daytime sleep-related behaviours among Israeli adolescents. Acta Paediatr. 99, 1396–1400. 10.1111/j.1651-2227.2010.01821.x 20377536

[B50] Singleton R. A. Wolfson A. R. (2009). Alcohol consumption, sleep, and academic performance among college students. J. Stud. Alcohol Drugs 70, 355–363. 10.15288/jsad.2009.70.355 19371486

[B51] Taras H. Potts-Datema W. (2005). Sleep and student performance at school. J. School Health 75, 248–254. 10.1111/j.1746-1561.2005.tb06685.x 16102087

[B52] Taylor D. J. Bramoweth A. D. (2010). Patterns and consequences of inadequate sleep in college students: substance use and motor vehicle accidents. J. Adolesc. Health 46, 610–612. 10.1016/j.jadohealth.2009.12.010 20472221

[B53] Voderholzer U. Piosczyk H. Holz J. Landmann N. Feige B. Loessl B.. (2011). Sleep restriction over several days does not affect long-term recall of declarative and procedural memories in adolescents. Sleep Med. 12, 170–178. 10.1016/j.sleep.2010.07.017 21256802

[B54] Wang X. Perry A. C. (2006). Metabolic and physiologic responses to video game play in 7-to10-year old boys. Arch. Pediatr. Adolesc. Med. 160, 411–415. 10.1001/archpedi.160.4.411 16585487

[B55] Wolfson A. R. Carskadon M. A. (1998). Sleep schedules and daytime functioning in adolescents. Child Dev. 69, 875–887. 10.1111/j.1467-8624.1998.tb06149.x 9768476

[B56] Wolfson A. R. Carskadon M. A. (2003). Understanding adolescent's sleep patterns and school performance: a critical appraisal. Sleep Med. Rev. 7, 491–506. 10.1016/S1087-0792(03)90003-7 15018092

[B57] Wolfson A. R. Carskadon M. A. Acebo C. Seifer R. Fallone G. Labyak S. E.. (2003). Evidence for the validity of a sleep habits survey for adolescents. Sleep 26, 213–217. 12683482 10.1093/sleep/26.2.213

[B58] Wood A. W. Loughran S. P. Stough C. (2006). Does evening exposure to mobile phone radiation affect subsequent melatonin production? Int. J. Radiat. Biol. 82, 69–76. 10.1080/09553000600599775 16546905

[B59] Wright K. P. Jr. Badia P. Myers B. L. Plenzler S. C. Hakel M. (1997). Caffeine and light effects on nighttime melatonin and temperature levels in sleep-deprived humans. Brain Res. 747, 78–84. 10.1016/S0006-8993(96)01268-1 9042530

[B60] Zunhammer M. Eichhammer P. Busch V. (2014). Sleep quality during exam stress: the role of alcohol, caffeine and nicotine. PLoS ONE 9:e109490. 10.1371/journal.pone.0109490 25279939 PMC4184882

